# Oral Fludrocortisone Test for Salt-Sensitive Screening in Hypertensive Patients: A Randomized Crossover Trial

**DOI:** 10.1155/2018/7437858

**Published:** 2018-11-15

**Authors:** Andrea Pio-Abreu, Isac de Castro, Giovanio Vieira da Silva, Katia Coelho Ortega, Decio Mion

**Affiliations:** ^1^Hypertension Unit, Renal Division of Hospital das Clínicas, University of São Paulo Medical School, São Paulo, SP, Brazil; ^2^Renal Division of Hospital das Clínicas, University of São Paulo Medical School, São Paulo, SP, Brazil

## Abstract

**Background:**

Salt sensitivity is associated with an increased cardiovascular risk, but the gold standard method (diet cycles) requires 24-h urine samples and has poor patient compliance.

**Objectives:**

Test the hypothesis that oral fludrocortisone (0.4 mg per day for 7 days) is a good alternative in identifying salt-sensitive patients.

**Methodology:**

We conducted a randomized crossover study with 30 hypertensive individuals comprising the following steps: (1) washout; (2) phase A (low- and high-sodium diet cycles); (3) washout 2; (4) phase B (fludrocortisone test). Phase A and B steps were performed in a random way. Consistent with the literature, we found that 53.3% were salt-sensitive according to the reference test. Using the ROC curve, the fludrocortisone test defined salt sensitivity by a median blood pressure increase of ≥3 mmHg. A good accuracy of fludrocortisone in detecting salt sensitivity was observed (AUC: 0.732±0.065; p<0.001), with 80% sensitivity and 53% specificity.

**Conclusion:**

The fludrocortisone test is a good option for screening salt sensitivity in hypertensive patients. However, the low specificity prevents this test from being an ideal substitute to the labor-intensive diet cycles exam in the definition of salt sensitivity. This clinical trial is registered with NCT01453959.

## 1. Introduction

Salt sensitivity, defined by a significant blood pressure (BP) increase in response to salt consumption, is a potential major health problem. A higher salt sensitivity has been reported in African Americans [1], the elderly [1, 2], obese individuals [3], and patients with high-sodium and low-potassium diet [4]. Previous studies revealed that approximately 51% of hypertensive and 26% of normotensive individuals are salt sensitive [5, 6]. This condition is associated with target-organ damage [6–9], impaired glucose metabolism [10], and higher rate of fatal and nonfatal cardiovascular events [11]. Based on this evidence, a recent Scientific Statement of the American Heart Association highlighted salt sensitivity as “an issue of clinical importance because the phenotype carries prognostic implications potentially as strong as those of traditional cardiovascular risk factors” [6].

In clinical practice, however, salt sensitivity is not measured due to significant challenges. Indeed, the current technique used for evaluating salt sensitivity comprises long diet cycles, good patient compliance with low- and high-sodium intake, significant costs associated with the formulation of standardized meals, and two 24-h samples for urine sodium [12–14]. Alternatives to the dietary cycle have already been proposed. One of the alternatives was proposed by Weinberger and colleagues [5]. These authors conducted a protocol in hypertensive and normotensive patients using an intravenous infusion of 2 liters of normal (0.9%) saline and after sodium and volume depletion that was induced by a low sodium diet (10 Meq) and furosemide administration [5]. However, De la Sierra et al. [15] found that the Weinberger test can lead to a significant classification error (>50%), even considering different BP cut-off points. Recent studies have proposed new methodologies, including those involving genetic tests, which have not been applicable until now [16–18]. Therefore, the need for a new diagnostic test is highly desirable.

Fludrocortisone acetate is a synthetic adrenocortical steroid with predominantly mineralocorticoid action. It acts in the distal renal tubules causing sodium retention [19]. A previous study from our group has revealed a marked decrease in sodium excretion on the fourth day of administration when patients were on their usual diet [20]. We believe that sodium retention caused by fludrocortisone could simulate the salt increase in the diet cycle test. Therefore, we tested the hypothesis that oral fludrocortisone administration at a dosage of 0.4 mg/day for 7 days can be used as an alternative to identify salt-sensitive patients instead of the traditional protocol.

## 2. Material and Methods

### 2.1. Participant Recruitment

This study was approved by the local Ethics Committee and registered under the CAPPesq protocol number 0343/10. All participants signed an informed consent form.

During a one-year period, lean and overweight adult hypertensive individuals aged 40–65 years from the General Hospital of the University of São Paulo Medical School, Brazil, were recruited in the study. Due to ethical reasons for performing antihypertensive withdrawal (see details ahead), we included patients with systolic BP (SBP) between 140 and 159 mmHg and diastolic BP (DBP) between 90 and 99 mmHg without taking antihypertensive medications or patients with controlled BP (<140/90 mmHg) in the presence of up to two classes of antihypertensive drugs. We excluded secondary causes of hypertension (except sleep apnea), diabetes mellitus, stage 3–5 chronic kidney disease (glomerular filtration rate of <60 ml/min/1.73 m^2^), previous stroke, coronary artery disease, peripheral vascular disease, heart failure, liver failure, use of drugs that could interfere with BP (e.g., nonsteroidal anti-inflammatory drugs, oral or injectable contraceptives, and corticosteroids), and pregnancy.

### 2.2. Study Design

**Figure 1:**
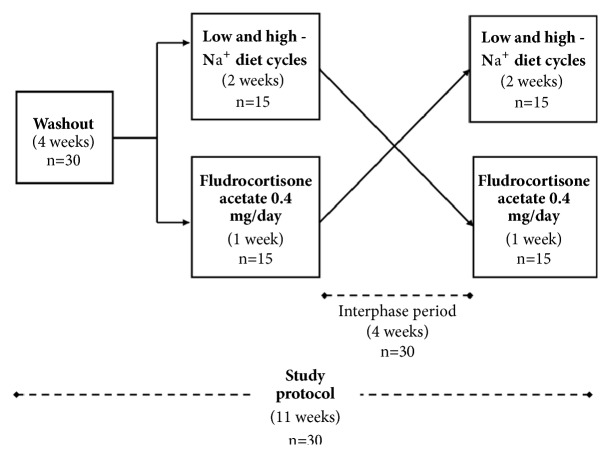
Study randomized crossover design. Na^+^ = sodium.

The study comprised the following steps: washout (medications withdrawn and no diet interventions), phase A (low- and high-sodium diet cycles), interphase period (washout 2), and phase B (fludrocortisone test). Using the four-block randomization system, patients were randomized into two groups. The randomization scheme was generated by using the web site Randomization.com (http://www.randomization.com). The first group followed the following order: washout 1, phase A, washout 2, and phase B. The second group was submitted to the following order: washout 1, phase B, washout 2, and phase A (Figure 1).

#### 2.2.1. Washout Periods

In the washout 1, all patients withdrew antihypertensive medications (until the end of the study). The washout 2 was devoted to avoid potential carryover effects from previous phases A or B. During these periods, BP was measured on the first visit with the validated Dixtal DX 2710 automated oscillometric device [21]. During these phases, patients consumed* ad libitum* diet (habitual diet without restrictions or interventions).

#### 2.2.2. Diet Cycles

The diets provided to the participants were controlled and produced by the same supplier (Condieta, São Paulo, Brazil). In addition to the two daily meals (lunch and dinner), participants were allowed to consume three servings of fruit a day and two servings of bread at breakfast. Bread without salt was provided for the low-sodium diet. The amount of sodium/day in the low- and high-sodium diet was ~40 and 200 mEq, respectively, promoting ~160 mEq sodium/day difference between the two regimens. On the first and seventh day of each cycle (phases A and B), patients were weighed and their BP was measured using the aforementioned automated method. After 10 min of rest in the supine position, 15 BP measurements separated by 2-min intervals were taken. We used the mean BP (MBP) value for analysis. In addition, 24-hour urine was collected for urinary sodium, potassium, and creatinine measurements. To avoid nonadherence to diets in the analysis, participants with urinary sodium values out of 10–50 mEq and 150–250 mEq ranges in the low- and high-sodium diets, respectively, were excluded.

As previously described, the criterion used for salt sensitivity was an increase of >5 mmHg in the MBP between the seventh day of a low sodium diet and the seventh day of high salt diet [16, 22–24].

#### 2.2.3. Fludrocortisone Test

All participants received 9-a-fluorocortisol (Florinef Acetate, ER Squibb & Sons), which was administered in the morning as a single oral dose of 0.4 mg per day for 7 days. During this phase, patients consumed their habitual diets. On the first and seventh days of fludrocortisone administration, patients were weighed and their BP was measured using the abovementioned automated method. The MBP cut-off value for identifying salt-sensitive patients in the fludrocortisone test was calculated based on the receiver operating characteristic (ROC) curve, corresponding to the observed point of maximal sensitivity and specificity. An ROC curve is a plot of sensitivity on the y-axis against (1 - specificity) on the x-axis for varying values of the threshold t. The area under the ROC curve is a summary measure that essentially averages diagnostic accuracy across the spectrum of test values [25].

Further, on the first and seventh days of tested medication, blood samples were collected for clinical analysis in order to check adherence to fludrocortisone. Serum and urinary sodium and potassium were obtained using the ion-selective electrode technique, and urinary creatinine was obtained using the colorimetric kinetic method by the Jaffe reaction. These analyses were conducted using an automated biochemical analyzer (COBAS 8000 model, Plus–Roche Diagnostic System, US). Furthermore, hemoglobin was obtained by the sodium lauryl sulfate method, and hematocrit was directly measured from the volume of each erythrocyte after impedance using an automated hematology analyzer (Sysmex XT-2000i model, Sysmex, Japan). Renin was measured using chemoimmunoassay with the DiaSorin kit (Minnesota, USA), and aldosterone was measured by radioimmunoassay using the Coat-A-Count aldosterone commercial kit (Siemens Healthcare Diagnostics Inc., California, USA).

### 2.3. Statistical Analysis

Continuous and semicontinuous data were compared with the Gaussian curve and categorized as parametric or nonparametric using the distance Kolmogorov–Smirnov (KS) and Shapiro–Wilk tests. We used Student* t*-tests and repeated measures analysis of variance with modified Tukey's test. The times during the low- and high-sodium diets were defined as the categories, and the continuous variable was defined as the difference between MBP values by subtracting BP on the seventh day of the low-sodium diet from BP on the seventh day of the high-sodium diet. We also used the Wilcoxon, Friedman, Muller–Dunn, and Chi-square tests. To determine the accuracy of the diagnostic test, we used the ROC curve. As previously described, the cut-off value for the fludrocortisone test was defined by the ROC curve. Significance was set at p<0.05.

## 3. Results

We initially recruited 96 volunteers. After excluding patients with BP ≥160x100mmHg, obese, elderly patients, and refusals, 34 patients were selected. After randomization, four additional patients were excluded because of a significant BP increase during the washout period (n=3) or lack of appropriate adherence to low sodium diet (n=1). The baseline characteristics of the 30 volunteers are reported in Table 1. Overall, we comprised overweight adult patients. Half of them were whites and 60% females.

### 3.1. Diet-Cycles Results

The average sodium excretion was 40±25 mEq/vol and 213±44 mEq/vol in the low- and high-sodium diet cycles, respectively. According to the criterion used for salt sensitivity (>5 mmHg MBP), 16 (53.3%) patients were classified as salt sensitive and the remaining (14 patients) as salt resistant.

### 3.2. Fludrocortisone Results

The mean sodium excretion, a measure of salt intake, during fludrocortisone administration was 187±84 mEq/vol. During the fludrocortisone test stage, there was a significant reduction in renin, aldosterone, potassium, hemoglobin, and hematocrit on both the fourth and seventh days of administration, whereas sodium remained stable (Tables 2 and 3). The fludrocortisone test did not promote significant changes in the aldosterone-renin activity ratio in the group of sensitive (11.8±6.0) and resistant (12.1±6.8) patients; p=0.30. Significant SBP (6.0±8.0 mmHg) and MBP (3.25±7.4 mmHg) increase were observed during this stage. At this stage, two volunteers (6%) reported dry mouth, with no other significant symptoms.

### 3.3. Diet Cycles vs. Fludrocortisone

There was no significant difference in the absolute increase in MBP between the first and seventh days of fludrocortisone administration (3.3±7.4 mmHg) and between low- and high-sodium diet groups (4.7±7.7mmHg). The ROC curve of the fludrocortisone test on the seventh day shows an AUC of 0.732±0.065 for MBP. The cut-off value to determine salt sensitivity by fludrocortisone test was established on the basis of the value with the highest sensitivity and specificity on the ROC curve, which was set at ≥3 mmHg increase for MBP. Using this cut-off, the test had a sensitivity of 80% and a specificity of 53% as compared to the gold standard >5 mmHg MBP by diet cycles.

## 4. Discussion

This randomized crossover study showed that the fludrocortisone test, which consists of administration of 9-a-fluorocortisol in the morning as a single oral dose for 7 days, had a good accuracy in identifying salt-sensitive hypertensive patients, as defined by a MBP increase ≥3 mmHg. Considering no need of 24-h urine samples and no salt restriction, fludrocortisone test seems to be a promising alternative screening method to identify salt sensitivity in patients with hypertension.

Based on the aforementioned importance of salt sensitivity in target-organ-damage and cardiovascular prognosis especially in the hypertension scenario [6], the development of feasible techniques may help to define salt sensitivity in the routine care. Our current practice relies on subjective reports from patients suggesting salt-sensitive profile which is not relevant or useful to the design and implementation of a personalized-based strategy of salt intake reduction [26]. In this sense, fludrocortisone test is pharmacologically sound, was well tolerated, and had minor side effects in our study. The one-week duration for this test was based on a previous study from our group [20]. We made attempt to test shorter periods (such as 4-day treatment) but the accuracy was not as good as 7 days (data not shown). Despite this, the current test spent 50% less time than the reference test [16, 22]. Previous studies of dietary salt sensitivity test have used different cut-off values according to the method of BP measurement [6, 16]. In general, the cut-off values are usually arbitrary and tend to be higher in studies using office BP measurements than in those using either automatic or ambulatory BP measurements [6]. It is important to highlight that we did not arbitrarily define or use the same definition from diet cycle for defining salt sensitivity in the fludrocortisone test. Instead, we defined the ≥3mmHg criterion based on the ROC curve, corresponding to the point of greatest sensitivity and specificity. The advantage of ROC analysis for quantitative diagnostic test is to determine the optimal cut-off points in ROC space [27, 28]. Although the fludrocortisone test has a good sensitivity of 80%, we acknowledge that this is not an ideal definitive test due to the low specificity (53%) but a good screening method. However, in the case of a screening test, it is recommended that it be fast and practical.

Our study has some strengths and limitations. This study used a randomized crossover design with washout periods. Baseline data from each phase suggest no carryover effects from the previous step [29]. Another study strength was the objective measurement of diet cycles and fludrocortisone adherence. Indeed, results from laboratorial exams (sodium, potassium, renin, and aldosterone) suggested the mineralocorticoid effects of the drug as a consequence of the good adherence to the test. The following limitations should be addressed: (1) Our study limited the salt sensitivity analysis to middle-age hypertensive patients with controlled or stage 1 hypertension. However, the reported frequency of salt sensitivity is consistent with the prevalence reported in several populations [5, 6, 16]; (2) like other salt-sensitivity techniques, all analyses were performed under no antihypertensive medications. Several drugs have obvious influences on sodium excretion and BP. Due to ethical reasons, we excluded patients with more severe forms of hypertension because we were not allowed to withdraw medications. Therefore, this test cannot be used in resistant hypertensives patients; (3) the cut-off point for salt sensitivity definition (5mmHg) was arbitrary in the reference test of the diets cycles. This cut-off was widely used in previous studies [16, 22–24] and described in the recent American Statement of Salt Sensitivity [6]; (4) an additional limitation is that we did not repeat the fludrocortisone test. However, the lack of significant differences in the results based on the order or randomization in our cross-sectional study may provide an indirect evidence of such reproducibility.

## 5. Conclusion

Our results suggest that fludrocortisone test seems to be a promising alternative screening method to identify salt sensitivity in patients with hypertension with no need of 24-h urine samples and no salt restriction.

## Figures and Tables

**Table 1:** **Demographic and laboratory data. **BMI = body mass index; SBP= systolic blood pressure; DBP= diastolic blood pressure.

**Parameter**	** Volunteers (n=30)**
**Sex**	60% female

**Age**	54±8 years old

**BMI**	26±3 kg/m^2^

**Ethnicity**	50% white
	36% black
	14% Asian descendants

**Office SBP on recruitment**	146±13 mmHg

**Office DBP on recruitment**	91±11 mmHg

**Serum creatinine **	0.8 ± 0.1 mg/dl

**Fasting blood glucose**	86 ± 4 mg/dl

**Table 2:** **Laboratory data during the fludrocortisone phase**. Na = sodium; K = potassium; Hb = hemoglobin; Ht = hematocrit; D1 Fludro, D4 Fludro, D7 Fludro = 1st, 4th, and 7th days of fludrocortisone, respectively. Data as mean ± standard deviation, compared by repeated measure analysis of variance followed by modified Tukey's.*∗* p<0.05 vs. D1 Fludro, † p<0.05 *vs.* D4 Fludro.

**Parameter**	**D1 Fludro**	**D4 Fludro **	**D7 Fludro**
**Na(mEq/l)**	142.6±2.3	142.4±2.4	143.1±2.2
**K (mEq/l)**	4.3±0.4	4.1±0.3*∗*	3.9±0.3*∗ ***†**
**Hb (g/dl)**	14.2±1.5	13.5±1.6*∗*	13.5±1.7*∗*
**Ht (**%**)**	42.0±3.5	40.4±3.9*∗*	40.0±4.1*∗*

**Table 3:** **Laboratory data during the fludrocortisone phase.** D1 Fludro, D4 Fludro, D7 Fludro = 1st, 4th, and 7th days of fludrocortisone, respectively. Data expressed as median and interquartile range (25th and 75th percentiles), compared by Friedman test followed by Dunn's test. *∗* p<0.05 vs. D1 Fludro.

	** D1 Fludro**			**D4 Fludro**			**D7 Fludro**		
**Parameter**	Median	**25**%	**75**%	Median	**25**%	**75**%	Median	**25**%	**75**%
**Aldosterone (ng/dL)**	8.6	7.4	12.5	4.9*∗*	2.5	7.1	3.6*∗*	2.6	6.7
**Renin (uUI/mL)**	6.5	3.6	15.6	3.3*∗*	2.0	6.0	3.0*∗*	1.6	6.0

## Data Availability

The clinical laboratory data used to support the findings of this study are available from the corresponding author upon request.
